# Synthesis of trace element bearing single crystals of Chlor-Apatite (Ca_5_(PO_4_)_3_Cl) using the flux growth method

**DOI:** 10.1186/1752-153X-7-56

**Published:** 2013-03-26

**Authors:** Stephan Klemme, Timm John, Mathias Wessels, Christof Kusebauch, Jasper Berndt, Arno Rohrbach, Peter Schmid-Beurmann

**Affiliations:** 1 Westfälische Wilhelms Universität Münster, Institut für Mineralogie, Corrensstrasse 24, Münster, 48149, Germany

## Abstract

We present a new strategy on how to synthesize trace-element bearing (REE, Sr) chlorapatites Ca_5_(PO_4_)_3_Cl using the flux growth method. Synthetic apatites were up to several mm long, light blue in colour. The apatites were characterized using XRD, electron microprobe and laser ablation ICP-MS (LA-ICPMS) techniques and contained several hundred μg/g La, Ce, Pr, Sm, Gd and Lu and about 1700 μg/g Sr. The analyses indicate that apatites were homogenous (within the uncertainties) for major and trace elements.

## Introduction

Apatite (Ca_5_(PO_4_)_3_(Cl,F,OH) is an ubiquitous accessory phase in igneous, metamorphic and sedimentary rocks. Natural apatites contain significant amounts of geologically relevant trace elements such as the rare earth elements (REE), high field strengths elements (HFSE) and large ion lithophile elements (LILE). Moreover, apatite is known to contain high concentrations of U and Th so that apatite formation can be established by conventional radioactive element decay dating or its thermal evolution can be reconstructed by investigating “fission tracks” caused by the decay of radioactive elements [1-5]. Furthermore, as human and animal bones consist of apatite, U-series dating of relatively young fossils is a new and exciting area of research in quaternary geosciences (e.g. [6]). To aid reliable analysis of trace element concentrations and isotopic ratios, matrix matched reference materials are needed. Single crystal homogeneous apatites that contain known amounts of trace elements would be ideal.

Moreover, apatite weathering and replacement processes in low-grade metamorphic rocks have been in the focus of research recently both in our institution and elsewhere [7-10]. This is mainly, as apatite, when equilibrated with or growing from a super-critical fluid in low-grade to high-grade metamorphic rocks, may contain a “geochemical fingerprint”, that is a trace element signature from which one might be able to re-construct the composition of the fluid. In order to calibrate such a fingerprint, experiments are needed to investigate the partitioning of trace elements between apatite and fluids in a range of chemical compositions, pressures and temperatures. The experiments in turn need well-characterized starting materials, i.e. trace element bearing homogenous single crystals of apatite.

Furthermore, phosphate ceramics have long been proposed as suitable materials for safe long-term nuclear waste storage [11,12]. Experiments to simulate interaction of such apatite-based ceramics with water-rich fluids [11,13-15] need suitable actinide-bearing apatite crystals as starting materials [16].

Here we report the high-temperature synthesis of mm-sized single crystal chlorapatites (Ca_5_(PO_4_)_3_Cl) using the so-called flux method. We tried several compositions, temperatures and synthesis routes and here we report on the most successful experiments, both in terms of crystal size as well as in terms of trace element homogeneity.

### Previous work

Several studies report the synthesis of single crystal apatite, both fluorapatite, chlorapatite and hydroxyapatite [17-23]. Most synthetic apatites contain no trace elements, only a few groups have synthesized apatites with high concentrations (ie. wt.%) of one or two REE [24,25]. Most synthesis routes involve hydrothermal synthesis at high pressure [26], especially when hydroxyapatite is involved.

## Experiments

**Figure 1:**
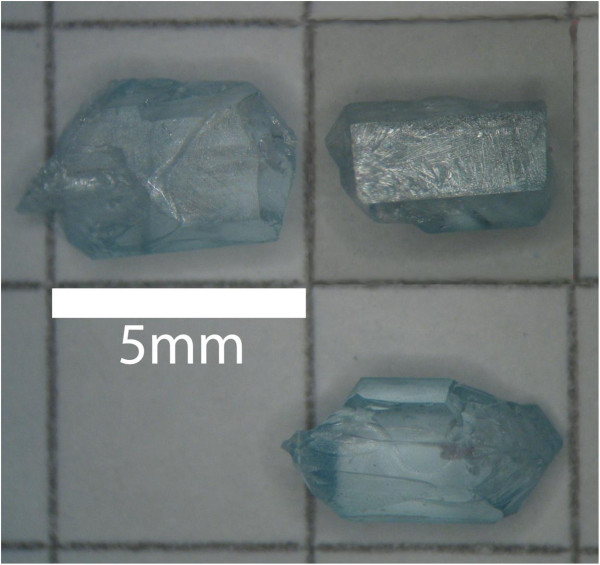
Chlorapatite crystals grown with the flux method; crystals from experiment SynCLAP6.

Initial experiments in chemical compositions without trace elements confirmed the validity of previous experimental results [23]. Using the flux growth method pioneered by Prener and others, we could grow idiomorphic apatite single crystals up to ca. 6 mm in size. All experiments were conducted in Pt-crucibles in conventional vertical high-temperature furnaces at atmospheric pressure. The starting material consisted mainly of various mixtures of Ca_3_(PO_4_)_2_ and CaCl_2_, the latter of which acted as the flux. The experiments were heated to a temperature above the liquidus, they were held for a short time, and then slowly cooled to a final run temperature. During the cooling apatite crystals formed from the melt. After quenching, the experimental products were washed in water or diluted HCl for several hours. This effectively removes all the CaCl_2_ flux. Table 1 lists experimental run conditions of each individual experiment. Figure 1 shows some representative single crystal apatites grown in our laboratory.

**Table 1:** Experimental run conditions

**Experiment**	**ST**	**RR**	**PT**	**H**	**CR**	**ET**	**Trace elements**
	**/**	**/**	**/**	**/**	**/**	**/**	
	**°C**	**°/h**	**°C**	**h**	**°/h**	**°C**	
SynCLAP3	800	70	1300	10	6	1100	No
SynCLAP5	800	70	1300	12	6	1025	No
SynCLAP6	800	70	1300	10	6	850	Yes
SynCLAP8	800	70	1420	10	6	800	Yes
SynCLAP9	800	70	1320	10	6	800	Yes
SynCLAP10	800	70	1370	20	6	800	Yes
SynCLAP11	800	70	1370	20	6	800	Yes
SynCLAP12	800	70	1370	20	6	800	Yes

ST: starting temperature, RR: ramp rate during heating to PT, PT: Peak temperature, H: hours at PT, CR: cooling rate down to ET, ET: final run temperature.

### X-ray powder diffraction (XRPD)

**Figure 2:**
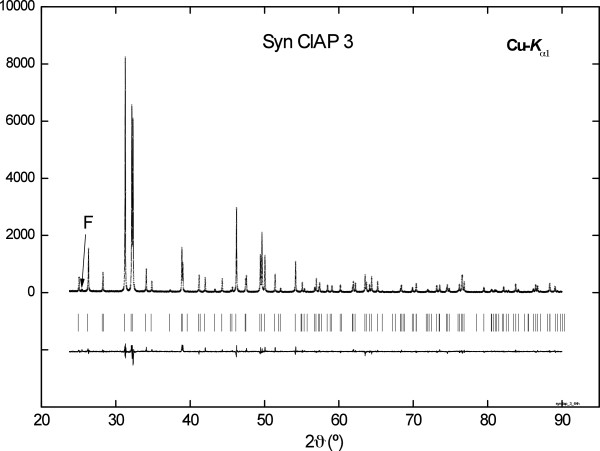
X-ray diffraction: Observed, calculated and difference intensity powder patterns of synthetic chlor-apatite.

For phase characterization an X-ray powder diffraction pattern was recorded using a PHILIPS X´PERT PW 9430 diffractometer with Cu-*K*_α1_ radiation and a primary Ge-(111) monochromator of Johansson Type. The operating conditions were 45 kV and 40 mA. Rietveld refinement was performed using the FULLPROF SUITE 2005 [27]. As starting parameters lattice parameters and crystal structural data including isotropic temperature factors for apatite-(CaCl) were taken from the literature [28]. The parameters which were varied for the refinement included the scale factor, the lattice parameters ***a*** and ***c***, 4 background parameters, the sample displacement, two asymmetry parameters as well as the shape parameters *w* and *Y* of the Thompson-Cox-Hastings pseudo-Voigt profile function. The refinement converged to an R_wp_ = 12.4% (R_exp_ = 9.4%). No significant line broadening could be detected with respect to the Si-640a NIST-Standard which was used to determine the resolution function of the diffractometer. As can be seen from Figure 2 one weak reflection at 25.41°(2θ) remained unexplained which is therefore assumed to belong to an additional unidentified phase. As its intensity is about 0.7% of that of the most intense apatite reflexion we assume that the amount of that phase is about 1% by weight. The results are given in Figure 2 and Table 2 together with recent literature data. In conclusion our apatite sample can be characterised as nearly pure chlor-apatite with very good crystallinity.

**Table 2:** **Unit-cell parameters of synthetic chlorapatites (space group *P6/3m*)**

	***Sample***	***a *[Å]**	***c *[Å]**
Chlor-Apatite			
This work	SynCLAP-3	9.6397(2)	6.7693(1)
García-Tuñón et al. 2012 [28]	Clap	9.6452(2)	6.7636(2)
Luo et al. 2009 [42]	THClAP	9.6330(2)	6.7834(2)
Luo et al. 2009 [42]	UClAP	9.6233(2)	6.7784(3)

### Synthesis of trace element bearing apatites

Once we were satisfied which the flux growth itself (SynCLAP3 and SynCLAP5, see Table 1), we conducted further experiments where the starting material contained a number of geochemically relevant trace elements. However, although we added relatively large amounts of trace elements (e.g., SynCLAP 6, 300 μg/g of each trace element, see Table 3) to the initial starting material mixture, we found that the resulting flux-grown apatites did not contain high concentrations of trace elements (generally well below 10 ppm of each trace element). We believe that the overall low concentrations of trace elements in the synthetic apatite crystals was caused by the fact that most of these trace elements, many of which are trivalent rare earth elements, are incorporated into apatites by a coupled substitution which involves incorporation of Na^+^ which replaces Ca^2+^ or of Si^4+^ which replaces P^5+^ in the apatite structure. Below we show two possible exchange mechanisms for the incorporation of trivalent rare earth elements (REE) into the apatite structure [29,30].

(1) $$Ca^{2+}+P^{5+}=\mathrm{RE}E^{3+}+Si^{4+}$$

(2) $$2\;Ca^{2+}=\mathrm{RE}E^{3+}+Na^{+}$$

**Table 3:** Starting materials

**Experiment**	**Ca**_**3**_**(PO**_**4**_**)**_**2**_	**CaCl**_**2**_	**Trace elements**
	**/**	**/**	
	**g**	**g**	
SynCLAP3	4.65	15.35	None
SynCLAP5	4.65	15.35	None
SynCLAP6	4.65	15.35	300 μg/g of REE, Sr, Y. Th, U, Pb, Ba, Rb, Li, B using the solution K-M1
SynCLAP8	1.16	3.84	3000 μg/g Sm added as Sm_2_O_3_, 2 wt.% Si added as SiO_2_ and 2 wt.% Na added as NaCl
SynCLAP9	1.16	3.84	Identical to SynCLAP8
SynCLAP10	1.16	3.84	3000 μg/g of La, Ce, Pr, Sm, Gd, Lu, Hf, Zr, Ta, Ti, Sc each (added as oxides) and 2 wt.% Si added as SiO_2_ and 2 wt.% Na added as NaCl
SynCLAP11	1.16	3.84	2000 μg/g of La, Ce, Pr, Sm, Gd, Lu each (added as oxides) and 2 wt.% Si added as SiO_2_ and 2 wt.% Na added as NaCl
SynCLAP12	1.16	3.84	1500 μg/g of La, Ce, Pr, Sm, Gd, Lu, Sr each (added as oxides) and 0.8 wt.% Si added as SiO_2_

K-M1: solution containing 1000 μg/g of several REE (La, Ce, Gd, Nd, Sm, Yb, Lu), Sr, Y, Th, U, Pb, Ba, Rb, Li, and B.

**Figure 3:**
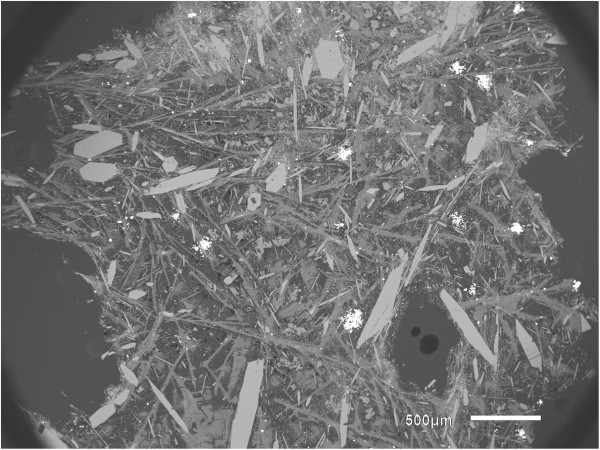
**SynCLAP10: Ideomorphic apatite crystals (lighter grey) in a matrix of acicular Ca-silicate crystals, most of it wollastonite (CaSiO**_**3**_**), after washing with HCl solutions.** The fine intergrowth of apatite with wollastonite needles makes physical recovery of apatite single crystals difficult.

We believe that the lack of Na^+^ and Si^4+^ in apatites grown in SynCLAP 6 strictly limited the incorporation of trivalent trace elements. Consequently, when we added some Na and Si (2 wt.%, SynCLAP 8, see Table 1 for details) to the starting material, we found that the flux-grown apatites contained significant amounts of Si and also significantly higher amounts of trace elements. This shows that incorporation mechanism (1) is more important than mechanism (2). Experiments SynCLAP 9 and 10 were similar to SynCLAP 8. The latter experiments yielded large and trace element bearing apatite but due to high SiO_2_ contents of the melt lots of other acicular, needle-like, Ca-silicates formed in the melt. It was difficult to separate apatite crystals from the quench-crystallized matrix after the flux had been washed out. Figure 3 shows typical textures observed in the experiments SynCLAP 8-10.

Consequently, SynCLAP 11 and 12 contained less REE and less Na and Si (Table 3). In conclusion, the apatite single crystal synthesis is best-done following procedures and compositions like in experiment SynCLAP 12. The apatite crystals grown in these experiments are large (see Figure 3), they contain high concentrations of trace elements (Table 4) and the apatite crystals can be easily removed from the matrix.

**Table 4:** Trace element concentrations in Apatites (SynCLAP12)

	1-1*	**1-2**	**1-3**	**1-4**	**1-5**	**1-6**	**1-7**	**1-8**
	**/**	**/**	**/**	**/**	**/**	**/**	**/**	**/**
	**μg/g**	**μg/g**	**μg/g**	**μg/g**	**μg/g**	**μg/g**	**μg/g**	**μg/g**
Mg	41	40	43	41	42	43	43	42
Si	10374	10186	10819	10118	9868	10847	10797	10630
Fe	4	4	6	7	8	5	9	4
Sr	1790	1696	1780	1660	1717	1674	1806	1669
La	867	844	887	845	868	1232	908	1085
Ce	16	16	17	16	16	20	17	19
Pr	574	573	598	567	583	823	599	717
Sm	552	552	595	546	556	824	584	722
Gd	548	548	594	537	547	832	584	725
Lu	100	98	108	98	96	125	107	119
	**2-1**	**2-2**	**2-3**	**2-4**	**2-5**	**2-6**	**2-7**	**2-8**
	**/**	**/**	**/**	**/**	**/**	**/**	**/**	**/**
	**μg/g**	**μg/g**	**μg/g**	**μg/g**	**μg/g**	**μg/g**	**μg/g**	**μg/g**
Mg	46	42	41	43	42	41	42	41
Si	10698	10377	10532	10679	10552	10600	11535	10605
Fe	18	3	6	7	7	7	8	9
Sr	1785	1722	1774	1666	1740	1705	1710	1760
La	888	816	853	831	814	844	838	1037
Ce	17	16	17	17	17	17	18	19
Pr	570	540	556	557	551	575	547	711
Sm	589	501	529	522	511	532	527	685
Gd	599	495	523	520	515	534	531	685
Lu	115	94	101	100	96	100	108	113
	**3-1**	**3-2**	**3-3**	**3-4**	**3-5**	**3-6**		
	**/**	**/**	**/**	**/**	**/**	**/**		
	**μg/g**	**μg/g**	**μg/g**	**μg/g**	**μg/g**	**μg/g**		
Mg	41	44	44	42	42	39		
Si	10508	12256	11634	11562	10897	10951		
Fe	<8.52	9	<8.17	<8.65	11	12		
Sr	1804	1819	1841	1851	1843	1706		
La	1008	884	851	869	915	1378		
Ce	20	21	19	18	19	23		
Pr	710	616	595	603	640	981		
Sm	706	598	567	585	623	1041		
Gd	681	561	533	548	584	985		
Lu	111	114	104	104	102	134		

Trace element analyses performed using Laser Ablation ICP-MS. Analytical uncertainties are in the order of 15%. *: The individual analysis numbers (e.g., 1-1 stand for crystal 1 analysis 1) correspond with numbers in white circles (SEM images) in Figure 3.

**Figure 4:**
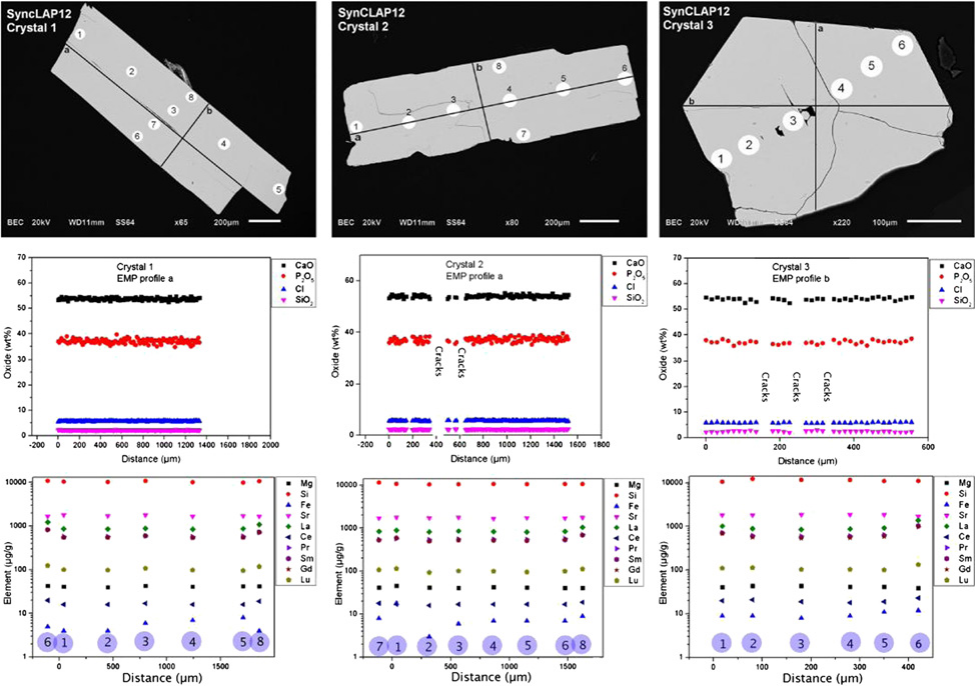
**Synthetic chlorapatite crystals from experiments SynCLAP12.** First row: back scattered electron images taken with an analytical scanning electron microscope (SEM). Crystals 1, 2 and 3 were analysed for major elements (Ca, P, Cl, Si) with electron microprobe analyzer (EMPA) and the black lines in the SEM pictures mark the line scans where EMPA Analyses were undertaken. The second row diagrams show the major element composition of the apatite crystals along the line scans. The third row diagrams show trace element concentrations of the apatite crystals which were analysed with Laser Ablation ICP-MS techniques at Münster University. The analyses are numbered (purple circles) and the analysis sites are given in the SEM pics in the first row.

### Trace element concentrations in synthetic apatites

When single crystals are grown from a melt (or flux), trace elements will be incorporated into the crystals. The concentration of the trace elements in the crystals depends on their equilibrium partition coefficients (if equilibrium is attained) and the bulk concentration of the trace element. If diffusion rates of trace elements are low in the crystal (and this is the case for all geologically relevant trace elements in apatite [31-33], crystals may be zoned, at least in elements which are compatible, that is elements with a crystal/melt partition coefficient >1. This is due to the fact that the first crystals formed will contain comparatively high concentrations of this compatible trace element and the coexisting melt will be consequently depleted in this element. Crystals that form later, or layers of the crystal which form later during cooling will contain significantly lower concentrations of the trace element. As it is well known that many REE, Sr and other important trace elements are compatible in apatite [29,34-37] we were concerned initially that our synthetic apatites may be significantly zoned. However, analytical results using in-house laser ablation ICP-MS techniques [7,36,38-41] show that the apatites synthesized in SynCLAP12 are rather homogeneous in terms of major and trace elements, surely within the analytical uncertainties. The homogeneity surprised us initially but this is probably due to the fact that the partition coefficients between apatite and CaCl_2_-rich flux are probably very different from the published apatite/silicate melt partition coefficients (e.g., [29]). Moreover, the flux/crystal ratio employed in our study is high which further minimizes potential zoning during crystal growth. Figure 4 shows major and trace element concentrations of some representative apatite crystals from SynCLAP12.

In summary, we present an effective procedure to synthesize mm-sized single crystals of chlorapatite that contain a variety of geochemically relevant trace elements. These crystals may be used as starting materials for further experiments or used as reference materials for geochemical analysis.

## Competing interests

The authors declare that they have no competing interests.

## Authors’ contributions

MW synthesized the samples, and together with JB and CK, performed the data analysis. SK drafted the manuscript; PSB carried out the XRD measurements and participated in the design of the experiments and helped to draft the manuscript. TJ, AR, and CK participated in the experimental design and coordination and helped to draft the manuscript. All authors read and approved the final manuscript.
